# Disorder-to-Order Transition in the CyaA Toxin RTX Domain: Implications for Toxin Secretion

**DOI:** 10.3390/toxins7010001

**Published:** 2014-12-31

**Authors:** Ana-Cristina Sotomayor-Pérez, Daniel Ladant, Alexandre Chenal

**Affiliations:** Institut Pasteur, CNRS UMR 3528, Unité de Biochimie des Interactions Macromoléculaires, Département de Biologie Structurale et Chimie, 28 rue du Dr Roux, Paris cedex 15 75724, France; E-Mail: acsotomayorperez@hotmail.com

**Keywords:** adenylate cyclase CyaA toxin, intrinsically disordered proteins (IDP), natively unfolded proteins, repeat in toxin (RTX), calcium-binding proteins, calcium-induced protein folding

## Abstract

The past decade has seen a fundamental reappraisal of the protein structure-to-function paradigm because it became evident that a significant fraction of polypeptides are lacking ordered structures under physiological conditions. Ligand-induced disorder-to-order transition plays a key role in the biological functions of many proteins that contain intrinsically disordered regions. This trait is exhibited by RTX (Repeat in ToXin) motifs found in more than 250 virulence factors secreted by Gram-negative pathogenic bacteria. We have investigated several RTX-containing polypeptides of different lengths, all derived from the *Bordetella pertussis* adenylate cyclase toxin, CyaA. Using a combination of experimental approaches, we showed that the RTX proteins exhibit the hallmarks of intrinsically disordered proteins in the absence of calcium. This intrinsic disorder mainly results from internal electrostatic repulsions between negatively charged residues of the RTX motifs. Calcium binding triggers a strong reduction of the mean net charge, dehydration and compaction, folding and stabilization of secondary and tertiary structures of the RTX proteins. We propose that the intrinsically disordered character of the RTX proteins may facilitate the uptake and secretion of virulence factors through the bacterial secretion machinery. These results support the hypothesis that the folding reaction is achieved upon protein secretion and, in the case of proteins containing RTX motifs, could be finely regulated by the calcium gradient across bacterial cell wall.

## 1. Introduction

**Figure 1:**
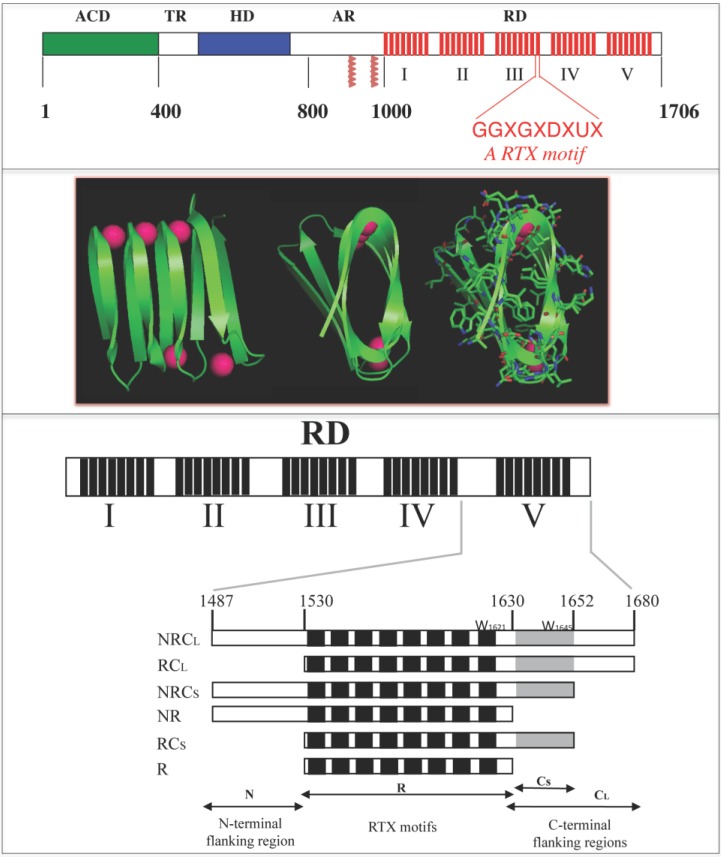
Schematic representation of the Repeat-in-ToXin (RTX) Domain from the adenylate cyclase (CyaA) toxin and crystal structure of the RTX domain from the Pseudomonas extracellular lipase (2Z8X). The upper panel shows CyaA that is made up of five main regions, the adenylate cyclase catalytic domain (ACD), the translocation region (TR), the hydrophobic region (HR), the acylation region (AR) and the *C*-terminal RTX domain (RD). RD is made of 5 consecutive blocks (I to V). Each red box represents a RTX motif. Native numbering of CyaA are given as rough indication and does not correspond to precise boundaries between CyaA regions. The middle panel shows snapshots of the β-helix of the RTX domain (residues 489 to 617) from the hydrolase crystal structure of the extracellular lipase from Pseudomonas (pdb entry: 2Z8X). The polypeptide is green and the calcium ions are magenta. A rotation of 90° is done from the left to the middle structure; the side chains are shown in the right figure to highlight that the core of the β-helix is made of hydrophobic residues while conserved aspartic acids are localized to the turns with calcium ions. The lower panel illustrates the organisation of the different RTX blocks of CyaA and the various polypeptides discussed here.

Disorder-to-order transition plays a key role in the biological function of many proteins that contain intrinsically disordered regions. This trait is exhibited by the RTX (Repeat in ToXin) protein family. Hereafter, we review recent works that provide a better understanding of the CyaA toxin RTX domain [1,2,3,4]. RTX proteins are widely distributed among Gram-negative bacteria. Despite the vast array of biological functions they can exert (they can be pore-forming toxins, metalloproteases, lipases, iron-regulating proteins, nodulation-related proteins, *etc*.) [5,6], they all have in common that they are secreted by the type 1 secretion machinery (T1SS) and they all require calcium to exert their biological functions [7]. RTX proteins receive this name due to the presence of RTX sequences that are glycine-and aspartate rich nonapeptides of the prototype GGXGXDXUX, where X stands for any amino acid and U any hydrophobic amino acid. RTX sequences are present in variable number (from 5 to more than 50) generally in a tandem fashion. X-ray crystallographic structure of the alkaline protease from *Pseudomonas aeruginosa* (an extracellular zinc-dependent protease) showed that RTX motifs fold in the presence of calcium into a characteristic calcium-binding structure: the parallel β-roll [8]. The parallel β-helix, or parallel β-roll, is a highly regular super secondary structure with calcium ions as an integral part [9]. The first six residues from the RTX motif (GGXGXD) form a turn involved in calcium binding while the last three residues (XUX) form a short β-strand, where the X residues point outwards and the U residue points inwards (it is buried in the core), contributing to the hydrophobic core of the β-helix (Figure 1). The conserved hydrophobic U residue stabilizes the hydrophobic core of the β-helix. Contrary to parallel β-sheets, the sheets of the central β-helix have only a slight twist, a consequence of calcium ions binding and also because the β-strand are only three residues in length [8,10]. The consecutive stacking of turns and β-strands build up a right-handed helix of parallel β-strands. This creates the parallel β-helix with calcium bound to both sides of the structure and with a narrow hydrophobic core in between. One turn of this helix consists of two consecutive RTX motifs (therefore, each GGXGXD motif forms two half-sites for calcium binding) and calcium ions are hexacoordinated between two spatially adjacent turns by the conserved aspartic acids and by the backbone carbonyl groups [8,9]. Since the RTX motifs bind calcium ions internally in the parallel β-helix, this structure is lost in the absence of calcium, due in part to electrostatic repulsion between negatively charged residues. Similar calcium-loaded β-roll structures have been found by X-ray crystallography in the *C*-terminal part of other RTX proteins, such as the metalloproteases from *Serratia marescens* and *Serratia sp.* [8,11] and by NMR in the R-module of the *Azotobacter vinelandii* calcium dependent mannuronan C-5 epimerase [12]. The structure at atomic resolution of RTX proteins in the absence of calcium has not been solved thus far due to its IDP behavior, as reviewed below.

One of the best-characterized members of the RTX family is the adenylate cyclase (CyaA) toxin from *Bordetella pertussis*, the causative agent of the whooping cough. CyaA is a 1706-residue long protein organized in a modular fashion [13,14]. The ATP-cyclizing, calmodulin-activated, catalytic domain (ACD) is located in the 400 amino-terminal residues [15,16] while the carboxy-terminal 1306 residues are responsible for the ACD translocation and the hemolytic phenotype of *B. pertussis*. Both activities can function independently as adenylate cyclase and hemolysin, respectively. Several domains can be identified in the *C*-terminal region. The so-called translocation region (TR), spanning residues 400 to 500, is crucial for the translocation of ACD across the plasma membrane and exhibits properties related to membrane-active peptides [17,18]. The hydrophobic region (HR), spanning residues 500 to 750, contains several hydrophobic segments predicted to adopt α-helical structures. The acylation region (AR), spanning residues 800 to 1000, contains two post-translational modification sites that are essential for the cytotoxic activities of CyaA. The toxin is indeed synthesized as an inactive precursor, proCyaA that is converted into the active CyaA toxin upon specific acylation of Lys 860 and Lys 983 by a dedicated acyltransferase, CyaC. The *C*-terminal part of CyaA is the cell receptor-binding domain (RD, residues 1000 to 1706). This domain consists of ~40 copies of a calcium-binding, glycine and aspartate-rich nonapeptide repeat that is characteristic of a large family of bacterial cytolysins known as RTX (Repeat-in-ToXin) toxins. These motifs constitute the main Ca^2+^ binding sites of the protein [19].

CyaA exerts a unique mechanism of translocation into host cells. It is able to translocate its catalytic domain directly across the plasma membrane of the target cell from the extracellular medium to the host cell cytoplasm where, upon activation by endogenous calmodulin, it increases the concentration of cAMP to supraphysiological levels that finally leads to the cell death [13,20]. Although the mechanism of translocation remains elusive, it is clearly established that calcium is absolutely required for the toxicity of CyaA. Indeed, calcium binding to CyaA is critical for the interaction of the toxin with the eukaryotic cell receptor, for the translocation of the catalytic domain inside the host cell and for the pore-forming activity of CyaA [21,22,23,24,25,26,27]. The RTX (RD) domain of CyaA is directly implicated in these calcium-dependent activities as it harbors the main calcium-binding sites of the toxins, the RTX motifs. The RTX motifs of RD are organized in five successive blocks (called from I to V). Each block contains 8–10 RTX motifs that are separated by non-RTX sequences of variable lengths (from 23 to 49 residues), which are called flanking regions (See Figure 1) [21]. Iwaki and co-workers previously showed that the last 217 *C*-terminal residues (encompassing the block V and its *N* and *C*-terminal flanking regions) constitute an autonomous domain required for both cytotoxic and hemolytic activities of the adenylate cyclase toxin [28]. Indeed, they showed that truncated CyaA proteins lacking the last 75 *C*-terminal residues did not exhibit any toxic activity. However, this defect could be complemented* in vitro* by any polypeptide having at least the last 217 residues of CyaA intact [28]. These results were further corroborated by Bejerano and co-workers, who showed that a stretch of 15 residues from the block V (residues 1636–1650) was crucial for the insertion of CyaA into the host cell membrane [29]. Both works suggested that the block V of CyaA played a key role in the intoxication process. It was later demonstrated that the flanking regions of block V were essential for calcium binding to the RTX repeat motifs [21]. Indeed, a polypeptide containing only the RTX motifs of block V (without flanking regions) was unable to bind calcium and its secondary structure was not affected by the presence of calcium. On the contrary, a polypeptide encompassing the RTX motifs of block V together with the *N*- and *C*-terminal flanking sequences was able to bind up to seven calcium ions per polypeptide with an affinity in the millimolar range. This polypeptide was also able to acquire secondary structure in the presence of calcium [21].

The involvement of the RTX motifs in calcium binding is clearly established. Therefore, the structural and functional consequences of calcium binding to these domains seem to play a crucial role in the understanding of RTX toxicity. Hence, understanding the conformational changes that take place in the RTX domains is important for the elucidation of the mechanism of calcium-dependent regulation of their biological functions. We review below a series of recent works that have contributed to provide a better understanding of the structure-function relationships of the RTX proteins [1,2,3,4]. In particular several CyaA RTX polypeptides of different lengths, ranging from 111 to 706 residues, were characterized and allowed to define key structural features implicated in calcium-induced folding of RTX proteins [1,2,3]. We further analyzed as a prototypical RTX polypeptide, a fragment of CyaA, RC_L_ that encompasses the last block of RTX repeat and that recapitulates most of the fundamental hydrodynamic, thermodynamics and structural properties of RTX proteins (See Figure 1).

We showed that the RTX proteins exhibit the hallmarks of intrinsically disordered proteins in the absence of calcium: they adopt pre-molten globule conformations and exhibit a strong time-averaged apparent hydration, due in part to the internal electrostatic repulsions between negatively charged residues, as revealed by the high mean net charge of the apo-polypeptide [4]. Calcium binding (2 mM CaCl_2_) triggers a strong reduction of the mean net charge, dehydration and compaction, folding and stabilization of secondary and tertiary structures of the RTX proteins. We propose that the intrinsically disordered character of the RTX proteins may facilitate their secretion through the bacterial type I secretion machinery (T1SS). These results support the hypothesis that the folding reaction is achieved upon protein secretion and, in the case of proteins containing RTX motifs, could be finely regulated by the calcium gradient across bacterial cell wall.

## 2. Calcium-Induced Disorder-to-Order Transition of RTX Proteins

**Figure 2:**
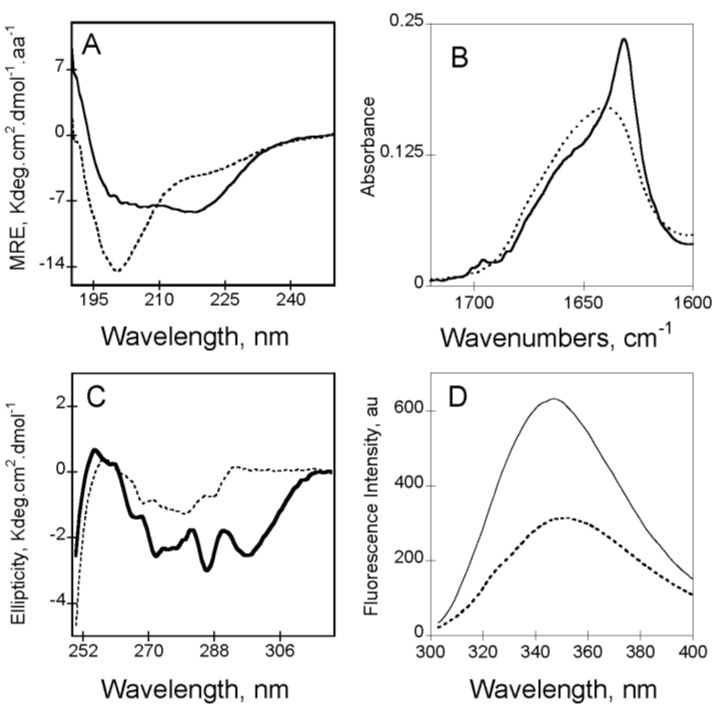
Calcium-induced conformational changes of RC_L_ (see polypeptide details in reference [4]) followed by circular dichroism in the far-UV region and near-UV region (Panels (**A**) and (**C**), respectively), followed by FTIR in the Amide I' region (Panel (**B**)) and followed by Tryptophan fluorescence spectroscopy (Panel (**D**)). In all panels apo-RC_L_ is represented with a dash line whereas holo-RC_L_ is showed with a continuous line. Experimental conditions: 20 mM Hepes, 100 mM NaCl, pH 7.4, ± 2 mM Calcium, see methods elsewhere [3,30].

In the absence of calcium (*i.e*., apo-state), RTX motifs are intrinsically disordered. Apo-RC_L_ exhibits a far-UV CD spectrum characteristic of an unfolded protein, with a strong negative π_0_–π* band around 200 nm characteristic of disordered conformations (Figure 2A). However, the presence of a weak negative n–π* band around 220 nm, suggesting the presence of some residual structures in the apo-state (Figure 2A). According to CD data, the FTIR-spectrum of apo-RC_L_ (Figure 2B) presents a wavenumber of maximum absorption typical of random coils (1645 cm^−1^). The FTIR spectrum was asymmetric, with a shoulder exhibiting weak absorption bands (between 1660 and 1680 cm^−1^) that could be attributed to a small proportion of turns and β-sheets. The tertiary structure of apo-RC_L_ can be investigated by CD and by tryptophan intrinsic fluorescence spectroscopy. The near-UV spectrum of apo-RC_L_ exhibited a large envelope of poorly resolved bands encompassing the aromatic region (Figure 2C). This result suggested the presence of residual tertiary structures (involving tyrosine and/or tryptophan side chains) that likely correlated with the weak secondary structure content suggested by the FTIR and far-UV spectra. In good agreement with the structural data obtained by CD, analyses of fluorescence experiments also indicate that the RTX polypeptides are mainly unfolded. In the absence of calcium, the maximum wavelength emission (λ_max_) of the RTX polypeptides was around 354 nm, indicating that the tryptophans in the apo-state were highly exposed to the solvent (Figure 2D). Moreover, analysis of ANS (8-anilo-1-napthalene sulfonic acid) binding did not reveal the presence of detectable solvent-exposed hydrophobic clusters in the apo-state, indicating that aromatic and aliphatic residues adopted disordered conformations. However, near-UV CD and 1D NMR data showed that few aliphatic and aromatic residues are in close proximity in the apo-state.

Beside the conformational studies, the investigation of the hydrodynamic parameters may help in determining whether a protein is compact or unfolded [31]. Using a combination of analytical ultracentrifugation, dynamic light scattering and size-exclusion chromatography coupled on-line to a tetra detector array (SEC-TDA) it is possible to determine the hydrodynamic properties of the RTX polypeptides [32]. Our results indicate that, in agreement with the characteristics described for IDP proteins, the RTX polypeptides exhibited higher hydrodynamic volumes than that expected for folded proteins of similar molecular mass (e.g., for apo-RC_L_, experimental *R*_H_ = 3.2 nm and expected *R*_0_ = 1.68 nm).

**Figure 3:**
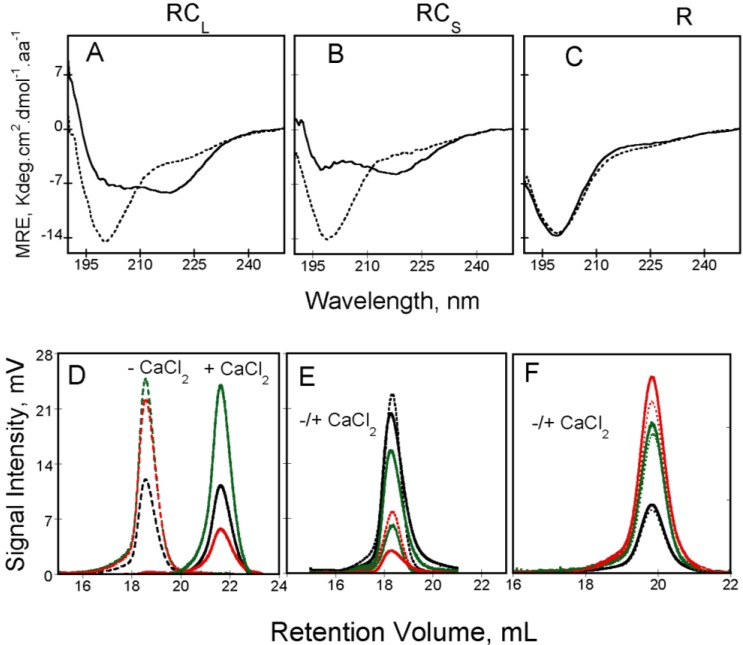
Calcium-induced conformational changes followed by circular dichroism in the far-UV region of RC_L_, RC_S_ and R polypeptides (Panels (**A**–**C**), respectively; see polypeptide details in reference [4]). Apo-RTX are represented with a dash line whereas holo-RTX is showed with a continuous line. Experimental conditions: 20 mM Hepes pH 7.4 100 mM NaCl, ±2 mM Calcium. (Panels (**D**–**F**)) show the behavior of RTX polypeptides on a Superdex 200 connected to TDA. RALS (**green** lines), differential refractometer (**black** lines) and differential viscometer (**red** lines) recordings were obtained for RC_L_ (**D**), RC_S_ (**E**) and R (**F**) polypeptides in the absence (dashed lines) or in the presence (continuous lines) of 2 mM calcium, see methods elsewhere [3,4].

Moreover, the RTX polypeptides in the absence of calcium, exhibited an unusually high intrinsic viscosity (13–15 mL/g), which in turn, dramatically affected their behavior in size exclusion chromatography (See Figure 3D). Interestingly, the high intrinsic viscosity is directly linked to the high hydration exhibited by the RTX polypeptides, which is about 10-fold higher than the one computed from its primary sequence (4 g/g* vs.* 0.4 g/g). Taken together, structural and hydrodynamic data indicated that the apo-RTX polypeptides exhibited the hallmarks of intrinsically disordered proteins.

To date, there are no three-dimensional data about the RTX motifs of CyaA. However, the X-ray structures of other RTX-containing proteins revealed that the RTX motifs fold in the presence of calcium in the so-called parallel β-helix (Figure 1). As described previously, in this structure, the first six residues (GGXGXD) of each RTX motif form a turn involved in calcium binding whereas the last three residues (XUX) of the motif form a short β-strands. Therefore, calcium ions are an important structural part of the parallel β-helix as the polypeptide chain folds around them [8,11]. No other divalent cation has been reported to induce such folding [3,21,33,34].

### 2.1. Conformational Changes Induced by Calcium Binding to the RTX Polypeptides

The addition of calcium induces the folding of the RTX polypeptides containing the *C*-terminal flanking region (See Figure 1 and Figure 3, and [3]). For instances, the analyses of the CD spectra, FTIR and fluorescence (Figure 2) revealed that, upon binding of calcium, the RTX polypeptides acquire both secondary and tertiary structures at the expense of disordered conformations. Secondary structure formation was revealed by the concomitant intensity decrease of the π_0_-π* and the increase of the n–π* band followed by CD in the far-UV region. Interestingly, careful analysis of the far-UV spectra of the RTX polypeptides in the holo-state (*i.e*., in the presence of calcium) reveals an original shape that does not exactly fit with the canonical spectra of α-helix or β-sheet structures. Indeed, the n–π* band presents a λ_max_ at 218 nm. This band could be a result of combination of the two n–π* bands characteristic of β-sheets (λ_max_ 216 nm) and α-helix (λ_max_ at 222) or, alternatively, it can be a unique band characteristic of parallel β-helix. Soon after the discovery of the parallel β-helix structure, Sieber and co-workers investigated whether this particular structure presents characteristic spectra in the far-UV region [35]. However, the experiments were not conclusive as the investigated proteins (pectate lyase PelC and PelB from *Erwinia chrysantehemi*) contain large non-RTX regions that can mask the contribution of this particular structure in the far-UV region of the CD spectrum. Working with proteins containing just the RTX motifs of CyaA (RD or the block V polypeptides), made it easier to decipher the specific contribution of the parallel β-helix structure to the CD spectrum. Several lines of evidence support that these unusual CD spectra of holo-RTX constitute indeed a particular signature of the parallel β-helix formed by the RTX motifs. First, the presence of an isodichroic point at 210 nm suggests that the binding of calcium controls the equilibrium between two conformations (between the apo-state and the holo-state). Second, the shapes of the far-UV CD difference spectra (resulting from the CD spectra at each calcium concentration minus the spectra in the apo-state) were similar at all calcium concentrations, suggesting that a unique structural motif was formed. Third, the far-UV CD difference spectra did not show negative bands around 206 nm characteristic of α-helical split π_0_–π* band. Fourth, the n–π* band at 218 nm was totally abrogated upon unfolding of the holo-state by high temperatures or chaotropic agents. Interestingly, similar spectra with a λ_max_ centered ~218 nm has also been found in proteins (or their fragments) made predominantly of RTX motifs, like in the lipase from *Pseudomonas* sp [36] or in the RTX domain of the α-hemolysin of uropathogenic *E. coli* [37]. In good agreement with the CD data in the far-UV region, the FTIR spectrum of the holo-state was dramatically different (see Figure 2B) with a major band located at 1627 cm^−1^ that could be assigned to β-sheets.

Similarly, addition of calcium induced significant changes in the tertiary structures of the RTX polypeptides as revealed by near-UV CD spectroscopy (Figure 2C). The near-UV CD bands located at 265 nm and 271 nm were typical of the weak negative and well-resolved vibronic bands from a phenylalanine L_b_ state, whereas the vibrational fine structures observed in the region from 275 to 282 nm may be assigned to overlapping tyrosine L_b_ bands and tryptophan L_a_ bands. Together, such near UV CD spectra revealed that several aromatic residues were involved in the formation and stabilization of the tertiary structures of the holo-state.

In all the calcium-binding RTX polypeptides, it was observed that the calcium affinity was strongly affected by the concentration of NaCl present in the buffer, suggesting that NaCl might compete with calcium for binding to the cation specific sites. However, NaCl *per se* did not affect the number of calcium ion bound to the RTX polypeptides and did not induce detectable changes in their far-UV CD spectra. Moreover, calcium-induced changes were found to be highly specific since no other divalent cation tested (Mg^2+^, Mn^2+^, Sr^2+^, Zn^2+^, Co^2+^, Rb^2+^ or Ni^2+^) was able to induce changes similar to calcium [3].

The calcium-induced folding was a highly cooperative process, with a Hill-number higher than five. This high cooperativity is particularly interesting if one consider the degeneracy of the RTX motifs of CyaA. Indeed, only half of the RTX motifs from block V matches with the canonical sequence GGXGXDXUX, the other ones missing one or several of the consensus residues. However, despite this degeneracy, the different RTX motifs of block V respond similarly to calcium in a cooperative manner. Cooperativity may arise because binding of several calcium ions cause the formation of several binding sites for the next ions (along the helical axis). In addition, ion binding on one side of the domain very likely strengthens the possibility for the turns of the opposite site to form and bind an ion [34]. This strong cooperativity could explain why mutations of the conserved aspartate residue in one of the RTX motif of the block V strongly reduced the overall affinity of the polypeptide for calcium ions [34]. In the experiments performed by Szilvay and co-workers, the aspartate residues of the fifth RTX motif (D_1570_) were mutated to an alanine or to a proline residue. They found a strong decrease in the overall calcium affinity of the modified protein (*K*_D_ varies from 0.15 mM to 0.85 mM), considering that only one RTX motif out of nine was altered. Furthermore, these authors observed that calcium binding by the modified polypeptide was not any more cooperative (with a Hill coefficient close to 1) [34]. The authors suggested that the mutation creates a nonresponsive site in the middle of the protein that could prevent cross talk between the two halves of the polypeptide chain and thus reduces the cooperativity [34]. However, the mutation performed by Szilvay and colleagues affected a RTX motifs located in the putative core of the parallel β-helix and it is possible that modification of aspartate residues within RTX motifs located at the extremity of the parallel β-helix could have a reduced effect on the structure.

Similarly to what was found for the block V polypeptides, the full RD domain of CyaA responses to calcium in highly cooperative manner. In the case of the full RD domain only 20 out of 40 motifs exhibit the canonical RTX sequence. It has been suggested that the non-canonical repeats may not coordinate calcium with the same affinity as canonical ones, and consequently, different affinities could be found for the different calcium-binding sites [38]. Surprisingly, this is not the case for RD, where the transition between the intrinsically disordered state and the folded one occurred in a narrow calcium concentration and with a Hill number higher than ten [2]. This suggests that the folding of the different blocks of RD occurs in a highly concerted manner and we propose that it could be initiated by binding of calcium to the RTX motifs of block V.

### 2.2. Hydrodynamics of Calcium Binding to the RTX Polypeptides

**Figure 4:**
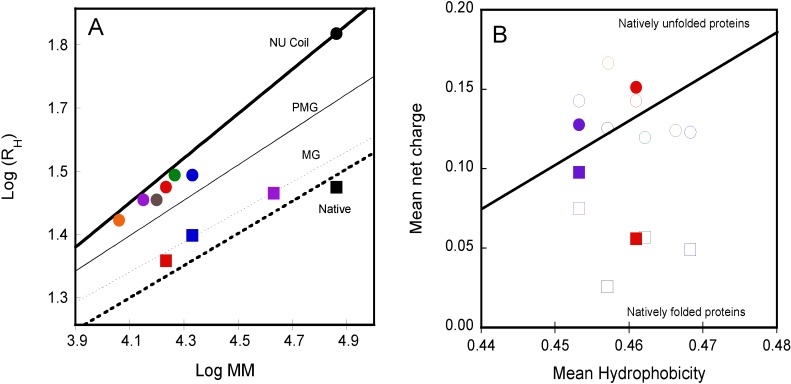
Dependency of the hydrodynamic radius on protein molecular mass (Panel (**A**)) and dependency of the mean net charge and the mean hydrophobicity of RTX polypeptides (Panel (**B**)). Both plots show the dependencies in apo-state (circles) and the holo-state (squares) of RD (black), NRC_L_ (blue), RC_L_ (red), NRC_S_ (green), RC_S_ (purple), NR (grey) and R (orange) polypeptides (see polypeptide details in reference [4]). In panel B, the filled symbols correspond to the mean net charge experimentally determined whereas the open symbols correspond to the theoretical mean net charge of the RTX polypeptides computed from their amino acid sequences; see [4] for details.

The hydrodynamic properties of the RTX polypeptides are also affected by calcium binding. The addition of calcium induces a strong compaction of the RTX polypeptides (for example, the *R*_H_ of RC_L_ decreases from ~3 nm in the apo-state to ~2 nm in the holo-state; see Figure 4A). This compaction is achieved because the positively-charged calcium ions are able to compensate the intramolecular electrostatic repulsions between the negatively charged aspartate residues in the apo-state. The introduction of calcium ions in the excluded volume of the polypeptides induces a reduction of internal charge repulsion, allowing the compaction of the protein due to the expulsion of water molecules from its hydrodynamic volume. The reduction of the protein hydrodynamic volume upon calcium addition fits very well with a disorder-to-order transition. Indeed after decades it is well known that there is a relationship between the molecular mass and the *R*_H_ of a protein depending on the polypeptide folding state. Figure 4A shows a graphical representation of the molecular mass-*R*_H_ dependency for IDP, pre-molten globule, molten globule and native proteins as compiled by Uversky and co-workers [39]. As seen in Figure 4A, the *R*_H_ values of the different RTX polypeptides in the apo-state lie between pre-molten globule conformations and natively unfolded coils, whereas the addition of calcium lead to a decrease of their hydrodynamic radii,* i.e*., a change along the *y* axis, to reach the region of compact proteins.

Additionally, the binding of calcium has also important consequences on the intrinsic viscosity of the RTX polypeptides. The hydrodynamic behavior of apo and holo-RTX was explored by SEC-TDA that allows simultaneous measurements of the molecular mass and the intrinsic viscosity of the polypeptides. Interestingly, apo-RC_L_ and holo-RC_L_ presented different retention volumes (see Figure 3D) although in both cases the molecular mass was similar and corresponded to that of the monomeric RC_L_. Therefore, the difference in the retention volume of both states arises from the unusually high intrinsic viscosity of apo-RC_L_. The high intrinsic viscosity of apo-RC_L_ explained its low retention volume on SEC and it is related to the hydrodynamic volume, which is proportional to the product of the molecular mass by the intrinsic viscosity, according to Einstein’s viscosity relation. Our analysis (combining data coming from AUC, QELS and SEC-TDA) revealed that addition of calcium induces a strong dehydration of the RTX protein: for instance, the hydration of RC_L_ decreases from 4.3 g/g in the apo-state to 0.90 g/g in the holo-state. Hence, this indicates that calcium binding triggered a massive dehydration of the polypeptide chain that folded into a compact state.

### 2.3. Thermodynamics of Calcium Binding to the RTX Polypeptides

Analysis of the thermal-induced denaturation of the RTX polypeptides monitored by tryptophan intrinsic florescence (rFI_360/320_) and by CD spectroscopy revealed that calcium binding induces a strong stabilization of the RTX motifs and that the structures lost by thermal denaturation corresponded mostly to the parallel β-helix fold formed upon calcium titration.

Indeed, temperature did not affect the weak n-π* band of the far-UV CD spectrum of apo-RTX, suggesting that the residual secondary structure in apo-state was very stable. On the other hand, the thermal-induced unfolding of the secondary structure of holo-RTX was characterized by the concomitant decrease of both the positive π_0_–π* band and the negative n–π* band. For high temperature, the far-UV CD spectrum of the denatured holo-state was similar to the spectrum of the native apo-state: a mostly unfolded polypeptide with residual secondary structure elements. The thermal-stability of the secondary structures at various calcium concentrations indicated that the stability of the secondary structure of RTX increased with the concentration of calcium. Similar results were obtained when near-UV CD signals were monitored as probe of the tertiary structures stability [2,3].

### 2.4. Reduction of the Mean Net Charge upon Calcium Binding to RTX Proteins

Electrostatic interactions play an important role in the disorder-to-order transition of IDPs, as observed here between RTX proteins and calcium. In the absence of calcium, the electrostatic repulsions between the numerous negatively charged Asp residues likely force the polypeptide chain to adopt extended and disordered conformations, whereas calcium binding to the RTX motifs partly neutralizes the Asp negative charges thus allowing the polypeptide chain to collapse and fold into a compact structure. Besides, the high sensitivity of holo-RTX to GdnHCl (the half denaturation of Holo-RTX occurred at GdnHCl concentrations around 0.3 M,* i.e.*, 10 fold lower than those of urea) suggest that the network of electrostatic interactions established between the calcium ions and the aspartic acids of RTX motifs is critical for the stability of the protein.

A direct experimental evidence of the charge effect on the unfolded-to-folded transition, was provided by the measurement of the electrophoretic mobility of the RTX proteins,* i.e.*, their velocities in a given electric field. In the absence of calcium, RC_L_ presents an electrophoretic mobility of −1.75 µm·cm·V^−1^·s^−1^ [4]. This electrophoretic mobility was converted into a number of charges by using the approach of Basak and Ladisch [40]. We thus determined that RC_L_ presents a mean net charge of −23 in the absence of calcium, a value similar to that computed from the primary sequence [4,41]. A large difference in electrophoretic mobility was observed between the apo- and the holo-state of the protein, as expected due to the binding of several calcium ions. Indeed, upon calcium binding, the electrophoretic mobility of RC_L_ changed from −1.75 to −1.06 µm·cm·V^−1^·s^−1^. This corresponds to a mean charge number of −23.6 for apo-RC_L_ and −9.1 for holo RC_L_. Moreover, the difference in the number of charges between apo-RC_L_ and holo-RC_L_ (−14.5) provides an estimation of the number calcium ions bound to the protein, assuming that the charge difference between the holo and the apo state is essentially due to the charge neutralization provided by the binding of this divalent cation. These data indicated that seven calcium ions were bound to holo-RC_L_, in good agreement with previous data suggesting that block V would bind six or seven calcium ions [21]. This approach was applied to several CyaA RTX constructions.

Uversky and co-workers [39] showed that mean net charge and hydrophobicity parameters allow separating intrinsically disorder protein from folded proteins (Figure 4B). The mean net charge determination placed the apo-RTX polypeptides in the intrinsically disordered region of the charge-hydrophobicity phase space, while the strong decrease of the mean net charge upon calcium binding shifts the holo-proteins well below the boundary between unfolded and folded proteins toward the folded space.

It is noteworthy that the mean hydrophobicity of the RTX proteins is off-centered to the set of intrinsically disordered proteins used to define the charge-hydrophobicity boundary. Indeed, the mean hydrophobicity of unfolded and folded proteins are 0.39 ± 0.05 and 0.48 ± 0.03 [42], respectively, while the mean hydrophobicity of the RTX proteins is 0.46 ± 0.05. This suggests that the hydrophobic forces are constitutive in the RTX proteins,* i.e.*, sufficient to drive the folding toward a native folded state. On the contrary, the mean net charge is high in the intrinsically disordered apo-RTX, favoring the unfolding. We propose that calcium binding decreases the repulsive electrostatic interactions within the intrinsically disordered proteins, allowing compaction, dehydration and folding. This transition between thermodynamic states is finely tuned by calcium binding and adapted to the physiological conditions. Indeed, the calcium concentration required for the folding of the RTX motifs coincides with the physiological calcium concentrations in the human serum and, moreover, the number of calcium ions bound to the RTX motifs allows the protein to switch from the intrinsically disordered to the folded area of the charge-hydrophobicity phase space. The mean hydrophobicity of the RTX proteins is close to that of folded proteins but off-centered compared to the set of intrinsically disordered proteins. These data highlight the fact that the calcium-induced mean net charge changes is the main trigger switching RTX proteins from disordered to ordered state.

## 3. The RTX *C*-Terminal Flanking Region Is Required for Folding

Functional complementation assays and biophysical analyses indicate that the polypeptide sequences flanking the RTX block V have an essential functional role in restoring the biological activities of truncated CyaA. Indeed, the hemolytic and translocation activities of a *C*-terminal truncated CyaA toxin can be restored by the addition of a protein corresponding to the lacking *C*-terminal RTX region. The RTX functional unit includes both the RTX motifs and the adjacent non-RTX flanking regions, which are essential for the folding and calcium responsiveness of the RTX domain [21]. To understand the contribution of the flanking regions to the RTX folding, six different polypeptides were constructed (Figure 1 and [3]), all of them comprising the core of the RTX motifs (R region, residues 1530–1630) but differing by the presence and/or absence of the *N*-terminal (N region, residues 1487–1529) and the *C*-terminal (C region, residues 1631–1680) flanking region. Based on secondary-structure prediction, the *C*-terminal region was further subdivided into two distinct segments: a short one, C_S_ (residues 1631–1652) and a long one, C_L_ (residues 1631–1680). The resulting polypeptides were named NRC_L_, RC_L_, NRC_S_, RC_S_, NR and R (see Figure 1).

The RTX polypeptides lacking the *N*-terminal flanking region (*i.e*., RC_L_ and RC_S_) were still able to fold in the presence of calcium (Figure 3A,B) indicating that this region is not required for calcium-induced changes. Comparison of the CD spectra of NRC_L_ and RC_L_ polypeptides suggest that the *N*-terminal flanking region remained unfolded both in the absence and in the presence of calcium [3]. This suggests that the *N*-terminal flanking region is probably not an integral part of block V folding unit and could rather serve as a tether with the upstream block IV of CyaA. However, it cannot be excluded that the *N*-terminal flanking region might also be directly involved in the folding of the preceding group of RTX motifs, that is, block IV. On the contrary, the *C*-terminal flanking region is essential for the calcium induced folding (See Figure 3). Indeed, RTX polypeptides lacking the *C*-terminal flanking region (NR and R polypeptides) were not able to fold in the presence of calcium and their hydrodynamic properties in the presence of calcium were similar to those observed in its absence: they were unfolded, flexible and highly hydrated.

Bejerano and co-workers described that a stretch of 15 amino acids encompassing residues 1636–1650 (called by the authors block A) was absolutely required for the insertion of CyaA into target cells. Indeed they showed that* in vitro* complementation of a CyaA protein truncated at position 1630 is achieved only in the presence of an RTX polypeptide containing the block A. Neither a short polypeptide composed of block A only nor a polypeptide consisting of eight RTX motifs, or a mixture of these two polypeptides, complements toxic activity [29]. Bejerano and co-workers suggested that homologous sequences to the so-called block A might be found in other RTX proteins. Additionally, Cortajarena and co-workers observed that the block A region was implicated in the interaction of another RTX protein, the hemolysin of *E. coli* (HlyA) with glycophorin, as HlyA mutant without block A were not able to bind erythrocytes [43]. Our structure-function studies on the calcium-induced folding of the CyaA RTX polypeptides described above provide a rationale for these observations. Indeed the critical role of “block A” in CyaA toxicity is likely due to the fact that these residues, as part of the *C*-terminal flanking region of block V, are absolutely required for calcium-dependent folding of CyaA. Hence, truncated proteins lacking block A sequence are not able to fold correctly and consequently, the misfolded protein can not exert its normal cytotoxic activity. Interestingly, the length of the *C*-terminal flanking region drastically influences the affinity for calcium. Data from calcium-titration experiments revealed that the polypeptides containing the short *C*-terminal flanking region (NRC_S_ and RC_S_) require much more calcium to acquire secondary and tertiary structures than those polypeptides having the long *C*-terminal flanking regions (compare for instances the apparent *K*_D _of RC_L_ = 0.26 mM with the apparent *K*_D_ of RC_S_ = 1.5 mM). Neither the long C_L_ nor the short C_S_ flanking regions were able *per se* to bind calcium and/or to acquire secondary structure upon the addition of calcium [3]. Moreover, an equimolar mixture of R and C_L_ polypeptides was not able to fold in the presence of calcium, suggesting that intramolecular tertiary contact, or proximity, between the C_L_ flanking region and the RTX motifs within a single polypeptide chain may be required to fold and stabilize the β-helix fold. Therefore, these data lead to the conclusion that the *C*-terminal flanking region plays a crucial role in the calcium-induced changes of the RTX polypeptides and that the length of the flanking region likely modulates the affinity of the RTX motifs for calcium [3,34]. However, the molecular basis allowing the *C*-terminal flanking region to induce the folding of the RTX motifs remains to be clarified.

Knowing the importance of the *C*-terminal flanking region in the calcium-induced folding of the RTX polypeptides, the hydrodynamic properties of the RTX polypeptides for which the length of the *C*-terminal flanking region is variable were investigated (Figure 4). Unexpectedly, the RC_S_ polypeptide (which is able to bind calcium and to acquire secondary and tertiary structure upon the addition of calcium) presented similar hydrodynamic radii (*R*_H_ ~3 nm) in the absence and in the presence of calcium. Moreover, both apo-RC_S_ and holo-RC_S_ presented similar retention volumes in size-exclusion chromatograph (Figure 3E). It was striking to see that an intrinsically disordered polypeptide could fold in the presence of calcium without noticeable changes of its hydrodynamic radii. Molecular mass analysis of our data indicated that the protein in the apo-state (14 kDa) corresponds to the expected mass of a monomer, while the molecular mass obtained in the presence of calcium (42 kDa) corresponds to a trimer. The results indicated that holo-RC_S_ eluted as a single peak of 42 kDa irrespectively of the protein concentration tested, demonstrating that the RC_S_ polypeptide folds upon calcium binding into a trimeric complex. The fact that no monomeric or dimeric calcium-bound intermediates could be detected suggests that holo-RC_S_ is only stable as a trimer [4]. The folding-pathway of RC_S_ is similar to the one found for RC_L_:RC_S_ binds calcium and folds acquiring both secondary and tertiary structures. Similarly to RC_L_, binding of calcium to RC_S_ triggers compaction and a decrease of the intrinsic viscosity due to the dehydration of the RTX motifs. However, the new feature is that the folding, compaction and dehydration of RC_S_ occur only upon the formation of a trimeric species (Figure 3E and Figure 4A).

These results suggest that the *C*-terminal flanking region does not only control folding and affinity but also the multimerisation state of the RTX motifs. Indeed, the propensity of the parallel β-helix to multimerize is not new. It has been suggested that intramolecular interactions within the β-helices are too weak to stabilize the structure in a monomeric state, but additional intermolecular interactions may provide enough energy to stabilize the assembly of β-helix proteins [9,44]. Several studies have indicated that intermolecular interactions may contribute to the stability of the β-helix structure. Some authors suggested that such a stabilization could be partially achieved by crowding agents such as polyethylene glycol [9,45]. About half of the known parallel β-helix proteins are trimers [46]. This observation could be fortuitous or related to the geometry of the β-helix structure. Furthermore, it is noteworthy that all these trimeric β-helix proteins are made of left-handed β-helix structures. Currently all reported RTX-containing β-helix structures are right-handed.

The fact that the β-helix structure *per se* is not thermodynamically favorable may also help to understand several observations reported here. The presence of the short *C*-terminal flanking region is not sufficient to stabilize β-helices in a monomeric protein. On the contrary, intermolecular interactions within a trimer of RC_S_ may provide enough energy to stabilize the β-helix structure. On the contrary, polypeptides containing the full-length *C*-terminal flanking region remain monomeric upon the addition of calcium. This feature could reflect a stabilization role of the long *C*-terminal flanking region, which may be able to provide enough interaction sites to stabilize a monomer of holo-RC_L_ through an entropic effect.

## 4. Role of the RTX Motifs in the Secretion of the CyaA Toxin

As described in the introduction, despite the vast array of biological functions that RTX proteins can exert, they all have in common that they are secreted by dedicated type 1 secretion machineries (T1SS). The fact that most RTX proteins are secreted by T1SS suggests that there might be a direct correlation between RTX containing proteins and their secretion by T1SS. There are more and more studies highlighting the importance of RTX motifs for efficient secretion through T1SS. First, deletions affecting the RTX motifs strongly affect the secretion of the truncated proteins. Indeed the removal of some RTX motifs from HlyA (α-hemolysin, an essential pore-forming RTX toxin produced by diverse uropathogenic *E. coli* strains) did not completely abolished but strongly affected the secretion of the toxin [47,48]. Similar results were found in the RTX lipase from *Pseudomonas* sp [49]. Secondly, it has been suggested that RTX motifs may act as an intramolecular chaperon. We demonstrated that the RTX motifs are unfolded at low calcium concentrations (similar to those present in the bacterial cytosol) [1]. The low intracellular concentration of calcium within the bacterial cell (0.1–1 µM) may prevent the folding of the RTX protein substrate and therefore favor the recognition of its secretion signal by the T1SS and its subsequent export through the narrow secretion channel.

**Figure 5:**
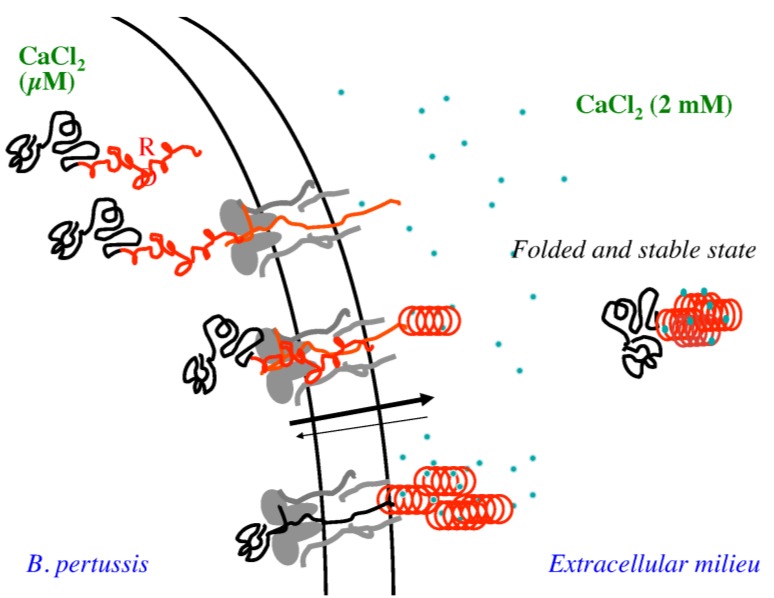
Schematic representation of the secretion of CyaA through the type 1 secretion system (T1SS). The RTX domain is highlighted in red, whereas calcium ions are blue.

Figure 5 presents a model for the secretion of RTX proteins suggesting that disorder-to-order transition that RTX motifs experienced upon calcium binding can play a key role in the secretion of all RTX-containing proteins. RTX motifs adopt intrinsically disordered pre-molten globule conformations in the low calcium environment of the crowded bacterial cytosol [1,30]. This unfolded conformation is partially due to the repulsion between the negatively-charged residues of the RTX motifs [4]. The high flexibility of the disordered states of the RTX motifs could facilitate the polypeptide uptake and transport across the narrow TISS secretion channel. Additionally, the negatively charged RTX sequences may be particularly favorable to harness the electrostatic field across the inner membrane as an energy source for the polypeptide export.

Assuming a directional secretion process of the polypeptide from its *C*-terminus toward the *N*-terminus, the block V should be the first block to exit the secretion channel into the calcium-rich environment of the bacterial extracellular medium. Calcium binding to the RTX motifs of block V might then trigger the folding of the RTX motifs into a compact and stable structure. The *C*-terminal flanking region would directly assist this folding. Indeed, we observed previously [3,4] that the full-length *C*-terminal flanking region of block V is mandatory for its stabilization. However, homologous sequences to the block V *C*-terminal flanking region cannot be found in the other four blocks of RTX motifs. It is then possible that the contribution of the *C*-terminal flanking region to the folding and stabilization is particularly important or exclusive for block V. Once block V is secreted and stabilized by calcium, it could serve as a nucleation site for the rest of the RTX region. Indeed, the folding of proteins containing repeated sequences has been frequently found to be initiated from a particular nucleation site [50,51]. Once block V is secreted, folded and stable, it might act as a nucleation site for the folding of the remaining blocks of RD. Such a cooperative reaction would improve the efficiency of the secretion process of the RTX-proteins. At present we do not know whether each block of the RD domain of CyaA folds independently or if they follow a sequential folding process initiated from block V. However, the fact that RC_S_ requires multimerization for structural stabilization likely suggests an inter-block stabilization among the five different blocks of the RD domain of CyaA. Finally, the difference of stability between the calcium-free disordered RTX polypeptide inside bacteria and the holo-state in the extra-cellular medium could likely constitute a significant driving force for the translocation of the whole protein through the secretion machinery. Once the RD domain is translocated and folded, it may interact with the CD11b/CD18 host cell receptor and it may also serve as a nucleus for the folding of the other domains of CyaA. It is noteworthy that disorder-to-order transitions in RTX toxins, and in proteins in general, are not only involved for secretion across bacterial membranes, but also for translocation across the host membranes to reach their cellular targets [52,53,54].
